# Immune Regulation of Mammary Fibroblasts and the Impact of Mammographic Density

**DOI:** 10.3390/jcm11030799

**Published:** 2022-02-02

**Authors:** Maddison Archer, Pallave Dasari, David Walsh, Kara L. Britt, Andreas Evdokiou, Wendy V. Ingman

**Affiliations:** 1 Discipline of Surgical Specialties, Adelaide Medical School, The Queen Elizabeth Hospital, University of Adelaide, Adelaide, SA 5011, Australia; maddison.archer@adelaide.edu.au (M.A.); pallave.dasari@adelaide.edu.au (P.D.); david.walsh@sa.gov.au (D.W.); andreas.evdokiou@adelaide.edu.au (A.E.); 2 Robinson Research Institute, University of Adelaide, Adelaide, SA 5001, Australia; 3 Peter MacCallum Cancer Centre, Melbourne, VIC 3000, Australia; kara.britt@petermac.org

**Keywords:** mammographic density, fibroblasts, breast cancer risk, immune signaling, stroma, extracellular matrix

## Abstract

Mammographic density is associated with a 4–6-fold increase in breast cancer risk independent of age and BMI. High mammographic density is characterized by breast tissue with high proportions of stroma comprised of fibroblasts, collagen, and immune cells. This study sought to investigate whether stromal fibroblasts from high mammographic density breast tissue contributes to increased extracellular matrix deposition and pro-tumorigenic signaling. Mammary fibroblasts were isolated from women with high and low mammographic density and exposed to immune factors myeloperoxidase (MPO), eosinophil peroxidase (EPO), transforming growth factor beta 1 (TGFB1) and tumour necrosis factor alpha (TNFA) for 72 h and profiled for expression of cancer-associated fibroblast and extracellular matrix regulation markers. No differences in gene expression profiles or collagen production were observed between fibroblasts with high or low mammographic density, and they did not have a differential response to immune mediators. MPO and EPO significantly increased the production of collagen 1. TGFB and TNFA induced variable changes in gene expression. Fibroblasts cultured in vitro from women with high mammographic density do not appear to be inherently different to those from women with low mammographic density. The function of fibroblasts in mammographic density-associated breast cancer risk is likely to be regulated by immune signals from surrounding cells in the microenvironment.

## 1. Introduction

Histological studies have reported that the key features of high mammographic density (MD) breast tissue are a higher abundance of stroma and greater deposition of collagen compared to low MD tissue [1,2]. Therefore, high MD breast tissue can be considered to resemble tissues with fibrosis, which are characterised by excessive deposition of collagen and extracellular matrixes (ECM) [3]. The most abundant cell type in the mammary stroma are fibroblasts, which are the key cell type responsible for fibrotic activities such as regulation of the synthesis and turnover of collagen and extracellular matrix (ECM) components [4,5]. High abundance of collagen associated with fibrosis increases breast cancer risk in a transgenic mouse model [6] and this suggests that fibroblast-mediated fibrosis could be a key driver of MD.

Fibroblasts can contribute to breast cancer risk and tumour development by acting as cancer-associated fibroblasts (CAF’s) (7). CAFs can be identified by their high level of expression of genes such as smooth muscle actin (*SMA*), vimentin (*VIM*), tenascin C (*TNC*), and fibroblast growth factor 5 (*FGF5*) [7,8]. CAFs can promote the transformation of normal epithelial cells to adopt more tumorigenic properties including increased invasion, motility and migration [9]. In breast cancer, CAFs express pro-inflammatory cytokines such as interleukin 6 and 8 to promote tumour cell proliferation and survival [10]. Further, these cytokines supress anti-tumour immunity during metastasis [11,12,13]. CAFs drive metastasis through remodelling of the ECM through expression of collagen genes, lysyl oxidase (*LOX*) and matrix metalloproteinases (*MMPs*) [11,12,13].

The activity of mammary fibroblasts can be influenced by signals from surrounding immune cells in the mammary stroma. Studies have reported that high MD tissue is associated with a more pro-inflammatory environment compared to low MD tissues and this is a driver of breast cancer risk [14,15]. Immune and inflammatory factors including peroxidase enzymes, transforming growth factor beta 1 (TGFB1) and tumour necrosis factor alpha (TNFA) have been shown to modulate some fibrotic and CAF functions of fibroblasts and play a role in other fibrotic diseases [16,17,18]. These factors have also been demonstrated to play roles in breast cancer [19,20,21,22]. How fibroblasts respond to these signals in high and low MD have not been previously investigated. We hypothesise that inflammatory immune factors that promote fibrotic and CAF related activities of mammary fibroblasts may be drivers of MD and breast cancer risk.

Currently, it is unknown whether fibroblasts are a driving factor for MD, if fibroblasts from women with high MD are inherently different in their behaviour to those from women with low MD, or if they respond differently to immune stimuli such as peroxidase enzymes, TGFB1 or TNFA. The aim of this study was to investigate whether fibroblasts derived from high MD breast tissue may be creating a favourable environment for tumour development through the expression of genes indicative of a CAF-like phenotype such as *SMA*, *COX2*, *FGF5*, *TGFB3*, *WNT5*, *VIM*, *TNC*, *IL6* and *IL8*. This study also aims to investigate the fibrotic activity of fibroblasts derived from high and low MD breast tissue by analysing the expression of genes involved in ECM regulation including *MMP1*, *MMP3*, *CTGF*, *LOX*, *FBN*, *COL1A1* and *COL4A5* as well as production of soluble collagen 1 and insoluble collagen. 

## 2. Materials and Methods

### 2.1. Study Population

**Figure 1:**
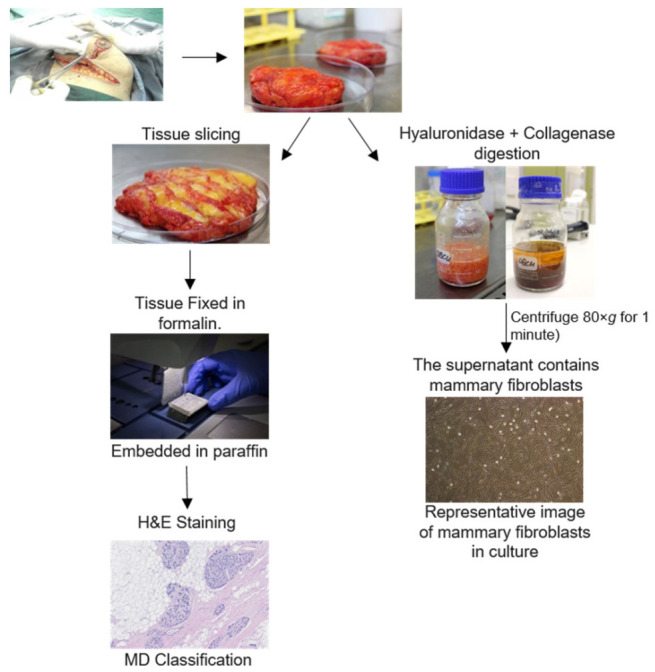
Protocol for breast tissue processing. Human breast tissues were collected from surgery patients. One portion of this tissue was enzymatically digested for 16 h. Mammary fibroblasts were obtained through several steps of washing the stromal cell component. Another portion of breast tissue was formalin fixed paraffin embedded and stained with haematoxylin and eosin for histological scoring of density.

This study was approved by the Human Ethics Committee at the University of Adelaide and The Queen Elizabeth Hospital (TQEH Ethics Approval #2011120). Between 2011 and 2017, participants undergoing reduction mammoplasty, mastectomy for breast cancer removal, or prophylactic mastectomy donated healthy breast tissue as confirmed by TQEH pathology department, a blood sample at the time of surgery, and completed a comprehensive medical and personal history questionnaire following surgery (*n* = 63). All participants were between the ages of 18 and 70 and were capable of giving informed consent. In the event that a participant was pregnant or currently undergoing chemotherapy, the participant was excluded from the study. The protocol followed for tissue collection is detailed in Figure 1. The total tissue collected was divided into two and one half undergoes enzymatic digestion for isolation of cells such as fibroblasts. The other half of the tissue is cut into fragments (approximately 1–2 cm^3^) and fixed and embedded in wax for histological analysis (Figure 1).

### 2.2. Tissue Embedding, Sectioning and Staining

Small fragments of breast tissue were dissected from each participant and fixed in 4% paraformaldehyde (Sigma Aldrich, St Louis, MO, USA; Cat#P6148) for 7 days at 4 °C. Tissue was processed using the Leica TP1020 Tissue Processor (Leica Microsystems, Wetzlar, Germany) moulded into paraffin wax blocks. Tissue sections were cut at 5 µm thickness using the Leica Rotary Microtome (Leica Microsystems, Wetzlar, Germany) onto SuperFrost Plus Slides (Menzel-Gläser, Braunschweig, Germany). Tissue sections were bonded onto slides by heating on a 37 °C heating block for 30 min.

### 2.3. Haematoxylin and Eosin Staining of Human Breast Tissue

Paraffin-embedded breast tissue sections were dewaxed using xylene (Merck Millipore, Darmstadt, Germany; Cat#108298) for 3 × 5 min and rehydrated in gradual dilutions of ethanol for 3 min each (2 × 100%, 1 × 90%, 1 × 70% and 1 × 50%) followed by 2 min in MilliQ water. Tissue sections were stained with haematoxylin (Sigma Aldrich, St Louis, MO, USA; Cat#HHS16) for 30 s then stained with eosin (Sigma Aldrich, St Louis, MO, USA; Cat#318906) for 10 s. Sections were then dehydrated through a gradual increase in ethanol concentration (2 min 90%, 2 × 1 min 100%), and cleared with xylene for 2 × 5 min. Slides were then mounted with coverslips using Entellan mounting media (Proscitech, Kirwan, QLD, Australia; Cat#IM022).

### 2.4. Histological Classification of Mammographic Density

**Figure 2:**
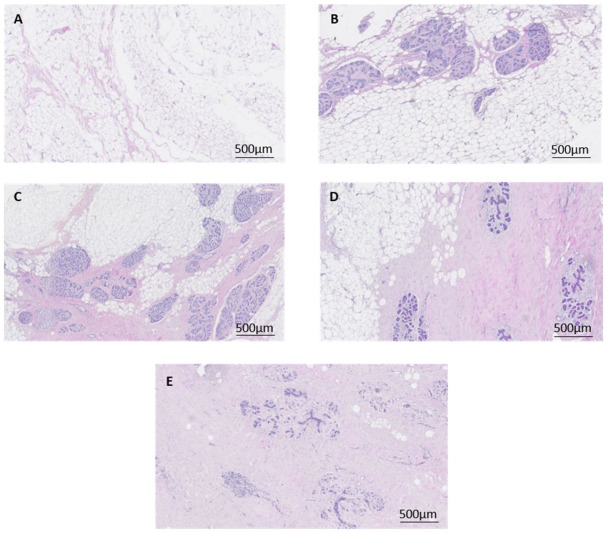
Histological classification of mammographic density. Haematoxylin and eosin-stained breast tissue was assessed by a panel of scientists to grade mammographic density according to the percentage area occupied by fibroglandular tissue. A density score of 1 is 0–10% (**A**), score of 2 is 11–25% (**B**), score of 3 is 26–50% (**C**), score of 4 is 51–75% (**D**) and score of 5 is greater than 75% fibroglandular tissue (**E**). These density scores were then used to select patient fibroblasts to be used in cell culture studies.

Haematoxylin and eosin-stained breast tissue sections were observed by a panel of 4 scientists (M.A., P.D., A.E., W.V.I.) to categorise each section on a semi-quantitative scale based on the percentage of fibroglandular tissue comprised of stroma and epithelium in relation to adipose tissue. One tissue section was assessed for each tissue block collected and a consensus on density was reached by the panel through discussion. A greater percent of stroma and epithelium corresponded to higher density scores. The classification scale ranged from 1–5 where 1 is 0–10%, 2 is 10–25%, 3 is 25–50%, 4 is 50–75% and 5 is >75% (Figure 2). This method has been shown to exhibit a reliable correlation with mammographic density determined by X-ray (1). Density scores for all the tissue sections of each patient were averaged to determine the overall fibroglandular density of the breast tissue. Tissue that scored between 1 and 2 was classified as low MD, tissue that was scored between 4 and 5 was classified as high MD. Tissue that scored between 2 and 4 was not classified as low or high and was not used in this study. All breast tissue samples that met the criteria for inclusion as high or low MD were used in the in vitro studies.

### 2.5. Isolation and Culture of Human Mammary Fibroblasts

A portion of participant breast tissue was designated for fibroblast isolation. This tissue is manually digested using a tissue knife then enzymatically digested using 100 U/mL hyaluronidase (Sigma Aldrich, St Louis, MO, USA; Cat#H3506) and 480 U/mL collagenase (Sigma Aldrich; St Louis, MO, USA, Cat#C0130) in Advanced Dulbecco’s Modified Eagle Medium/F12 (Life Technologies, Scoresby, VIC, Australia; Cat#12634010) supplemented with 10% foetal calf serum (FCS) (Thermo Fischer Scientific, Scoresby, VIC, Australia; Cat#10099141), 10 mM HEPES (Life Technologies; Scoresby VIC, Cat#15630080), 2.5 mg/mL Fungizone (Life Technologies; Scoresby, VIC, Australia Cat#15290018), 1 × L-Glutamine (Life Technologies; Scoresby, VIC, Australia, Cat#25030081) and 1 × Penicillin/Streptomycin (Life Technologies; Scoresby, VIC, Australia Cat#15240062). The tissue digestion flask was sealed using parafilm and was incubated overnight for 16–18 h in a 37 °C shaking water bath. The digest was then centrifuged at 80× *g* for 1 min and the top liquefied fat layer carefully removed and discarded. The supernatant containing stromal cells was carefully harvested so as to not disturb the pellet of undigested tissue then transferred to a clean Falcon tube and the pellet was then re-suspended and brought to 50 mL with supplemented Advanced DMEM/F12. The product was then centrifuged at 400× *g* for 5 min, supernatant was discarded, and the pellet re-suspended in 40 mL of supplemented Advanced DMEM/F12 medium. This was repeated until the supernatant became clear. The stromal cell pellet was treated with red blood cell lysis buffer (BD Bioscience, San Jose, CA, USA; Cat#555899) for 15 min, equilibrated with PBS, and filtered through 40 µm cell strainers (Sigma Aldrich; St Louis, MO, USA Cat#CLS431750) to remove debris. The cell suspension was washed 3 times in PBS by centrifugation at 400× *g* for 5 min and the supernatant discarded. The pellet was re-suspended in 8 mL of supplemented Advanced DMEM/F12 with 10% heat inactivated foetal calf serum, cultured in a T25 tissue culture flask and maintained at 37 °C in 5% CO_2_. Mammary fibroblasts were confirmed by morphological appearance in the culture and production of collagen 1.

### 2.6. Cytokine and Peroxidase Treatment in Human Mammary Fibroblast Culture

Mammary fibroblasts were selected for cell culture according to their histological mammographic density score. For these studies, fibroblasts were used from women with low density scores (1), and high density scores (4 and 5) (Figure 2). Mammary fibroblasts from human participants were cultured with supplemented Advanced DMEM/F12 containing 10% heat-inactivated FCS in a T25 tissue culture flask to 90% confluence. The cells were then split into 5 × T75 tissue culture flasks to 90% confluence. Cells were then distributed into 2 × 6 well plates at a density of 2 × 10^5^ cells per well and 1 × 96 well plate at a density of 1.2 × 10^5^ cells per well and grown to 90% confluence in supplemented Advanced DMEM/F12 with 10% FC. Cells were then serum starved overnight in serum-free supplemented Advanced DMEM/F12. Cells were treated for 72 h with either TGFB1 (10 ng/mL) (R&D Systems, Minneapolis, MN, USA; Cat#240-B-010), eosinophil peroxidase (2.5 µg/mL) (Lee Biosolutions, Maryland Heights, MO, USA; Cat#342-60), myeloperoxidase (5 µg/mL) (R&D Systems; Minneapolis, MN, USA, Cat#3174-MP-250), TNFA (10 ng/mL) (Life Technologies; Scoresby, VIC, Australia Cat#PHC3016) or CCL2 (500 µg/mL) (R&D Systems; Minneapolis, MN, USA, Cat#RDS27MC050) in the presence or absence of 100 µmol/L ascorbic acid (Wako Chemical Industries, Osaka, Japan; Cat#50990141) in serum-free supplemented standard DMEM medium (Life Technologies; Scoresby, VIC, AustraliaCat#12430054).

### 2.7. Enzyme-Linked Immunosorbent Assay

Soluble collagen type I in cell conditioned media was measured using a direct coat enzyme-linked immunosorbent assay (ELISA). Standard curves were developed using purified collagen I derived from human placental collagen (BD Biosciences, Australia; Cat#354265). Cell supernatants and standardised samples were added to a 96-well Maxisorp plate (Nunc, Roskilde, Denmark; Cat#423501) at a volume of 100 μL per well and stored at 4 °C overnight. The plate was washed with PBS-Tween 0.05% and blocked using a solution of 2.5% bovine serum albumin (BSA) in PBS for 1hr at room temperature. PBS-Tween was used to wash between steps for collagen ELISA for effective washing without disrupting the collagen–antibody binding. The primary antibody used was rabbit anti-human collagen I polyclonal antibody (Rockland Immunochemicals, Pottstown, PA, USA; Cat#600-401-103-0.5) and was added to the plate at 0.25 μg/mL in a 5% skim dairy milk solution for 3 h at room temperature with agitation. After washing, the secondary antibody europium-tagged anti-rabbit antibody (Perkin Elmer Life Sciences, Turku, Finland; Cat#AD105) was added at 0.5 μg/mL in 1% BSA/PBS for 1 h at room temperature. After the plate was washed, an enhancement solution (Perkin Elmer Life Sciences, Turku, Finland; Cat#1244-105) was added for 15 min before measuring fluorescence using the FLUOstar Optima plate reader (BMG Labtech Australia, Mornington, VIC, Australia) at excitation 355 nm and emission 620 nm. The collagen concentration for each sample was determined using the standard curve and expressed in µg/mL.

### 2.8. Sirius Red Analysis of Insoluble Collagen

Sirius red dye was used to determine changes in insoluble collagen deposition. Media was aspirated from fibroblast culture plates and washed in PBS. Cells were fixed for 10 min using 100% ethanol. The plate was washed under running tap water and picric acid–sirius red solution (Sigma Aldrich, St Louis, MO, USA; Cat#365548, Cat#P6744) was added for 1 h. After the stain was removed, the plate was washed with 0.01 M hydrochloric acid and allowed to air dry. To elute the dye, 0.1 M sodium hydroxide was added to the plate. Absorbance was then measured on the FLUOstar Optima plate reader (BMG Labtech Australia, Mornington, VIC, Australia) at 550 nm.

### 2.9. RT-qPCR Analysis

After 72 h culture, RNA was then isolated using RNeasy Mini Plus Kit (QIAGEN, Dandenong, VIC, Australia; Cat#74104) as per the manufacturer’s instructions. RNA was measured using 1 µL of sample on the Spectrophotometer ND 1000 Nanodrop using the program ND-1000 V3.7.1. The iScript cDNA synthesis kit was used to reverse transcribe all RNA samples into cDNA (Bio-rad technologies; Hercules, CA, USA Cat#1708890) according to the manufacturer’s instructions. Each cDNA reaction contained 500 ng of RNA from cell lines. Samples were incubated on a thermocycler for 5 min at 25 °C, 30 min at 42 °C and 5 min at 85 °C. cDNA was then diluted 1:10 in nuclease-free water to a volume of 200 µL. Quantitative real time PCR (qRT-PCR) was performed to determine the mRNA expression of genes of interest. Reaction mixtures were prepared in a volume of 10 µL, each containing 5 µL of SYBR green master mix (Bio-Rad technologies, Hercules, CA, USA), 0.4 µL of each forward and reverse primer, 2.2 µL of nuclease free water and 2 µL of sample cDNA. Reactions were run on a thermocycler using CFX96 Real Time Detection System running CFX Manager 3.0 software (Bio-Rad technologies; Hercules, CA, USA). The cycle conditions were 3 min at 95 °C, 40 amplification samples of 15 s at 95 °C, 15 s at 60 °C and 30 s at 72 °C. Each PCR plate contained no template controls of each gene, and all samples were run in triplicate. Data were analysed by the comparative Ct method relative to a housekeeping gene (HPRT1). The results were normalised to housekeeping gene expression and normalised such that the average of the control samples was 1 (∆∆CT). All primers were obtained from Geneworks (Thebarton, SA, Australia).

### 2.10. Statistical Analysis

Statistical analysis was performed using SPSS software, version 20.0 for Windows (SPSS, Chicago, IL, USA). Data were considered statistically significant when *p* < 0.05. An asterisk (*) identifies a result that is statistically significantly different from the control. All cell culture data, including RT-PCR and collagen production experiments, are presented as mean ± SEM (standard error of mean). Statistical analysis of RT-PCR data was performed using ∆CT values. These data were analysed using a Linear Mixed-effects Model to account for variables such as patient density and treatment groups. pPost hoc comparisons were performed to determine statistically significant differences in mRNA expression or collagen production between patients with high or low mammographic density and then between cells treated with immune regulatory proteins and untreated controls.

## 3. Results

Primary mammary fibroblasts were isolated from women with MD classified as low (*n* = 9) or high (*n* = 7) as identified by histological density categories (Table 1). In agreement with well-established inverse correlations of MD with age and body mass index (BMI), participants with low MD were older and overall exhibited a higher BMI than participants with high MD. Two thirds of participants with low MD had breast cancer, and most of the participants in the high MD cohort did not have breast cancer. The majority of participants were parous. None of the participants carried a *BRCA* mutation; however, a few had family history of breast cancer.

### 3.1. CAF and ECM mRNA Expression and Collagen Production of Mammary Fibroblasts from Women with High and Low MD

To investigate whether mammary fibroblasts are inherently different if they are derived from patient breast tissue with high or low MD, primary mammary fibroblasts were cultured for 72 h in serum-free media. mRNA expression of CAF-related genes and genes involved in ECM regulation were measured using quantitative real-time PCR relative to the expression of the housekeeping gene HPRT1 from the same patient. Results are expressed in arbitrary units as the data were normalised so that the average expression of each gene in untreated controls was equal to 1.

**Figure 3:**
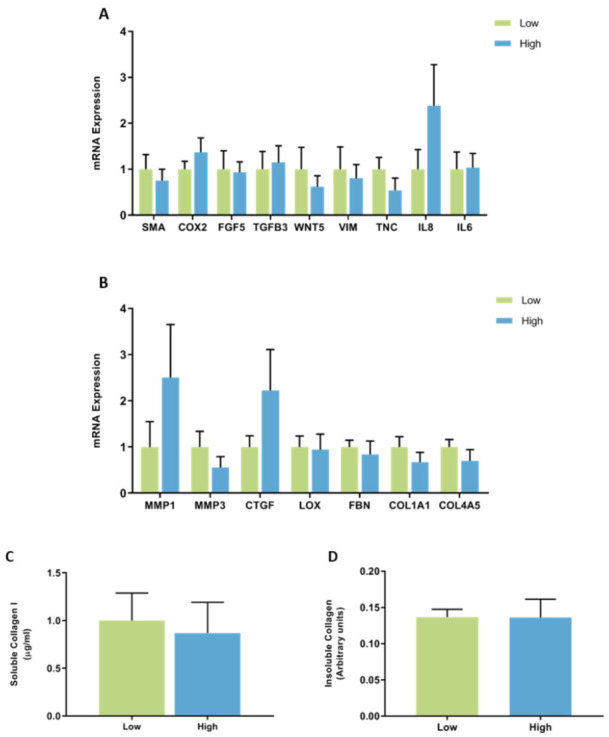
mRNA expression and collagen production in mammary fibroblasts isolated from women with high and low mammographic density. Messenger RNA expression of cancer-associated genes (**A**) and genes involved in extracellular matrix regulation and collagen production (**B**) measured by RT-PCR in human mammary fibroblasts from women with high (*n* = 7) and low (*n* = 9) mammographic density. mRNA expression normalised to HPRT1 expression and presented as relative expression where the average for untreated control is 1. Collagen production was measured using collagen 1 ELISA (**C**) and insoluble collagen measured by sirius red staining (**D**). Data presented as mean + SEM with statistical analysis performed using a linear mixed-effects model Tukey’s post hoc comparison.

No significant differences in the expression of CAF genes were observed between fibroblasts from women with high or low mammographic density. mRNA expression of IL8 had a large amount of variability between patient samples ranging from 0.10 to 5.42 arbitrary units in fibroblasts from high MD tissues (Figure 3A). No significant differences were observed in the mRNA expression of genes involved in ECM regulation between high and low MD mammary fibroblasts and there was a large amount of variation in the expression of MMP1 (0.08 to 8.83 arbitrary units) between high MD patient samples (Figure 3B).

Collagen production was measured by ELISA to detect the production of soluble collagen I and Sirius red dye to measure insoluble collagen fibres. No significant differences were seen in collagen production between fibroblasts from women with high or low MD (Figure 3C,D).

These studies suggest that fibroblasts from high MD breast tissue are not inherently different from those derived from low MD. However, fibroblasts are responsive to signals in the surrounding environment that can affect fibroblast function and may differentially stimulate their activity dependent on whether they were derived from tissue of high or low MD. Therefore, we investigated the effects of immune regulatory proteins MPO, EPO, TGFB and TNFA on mammary fibroblast activity and whether the response is dependent on whether fibroblasts were derived from high or low MD breast tissue.

### 3.2. The Effect of MPO on CAF and ECM mRNA Expression and Collagen Production by Mammary Fibroblasts

To investigate the effects of MPO and mammographic density on mammary fibroblasts, primary mammary fibroblasts were cultured for 72 h with 5 µg/mL of MPO in serum-free media (*n* = 16). mRNA expression of CAF-related genes and ECM regulation were measured using quantitative real time PCR relative to the expression of the housekeeping gene HPRT1 from the same patient. Results are expressed in arbitrary units and the data were normalised so that the average expression of each gene in untreated controls was equal to 1.

**Figure 4:**
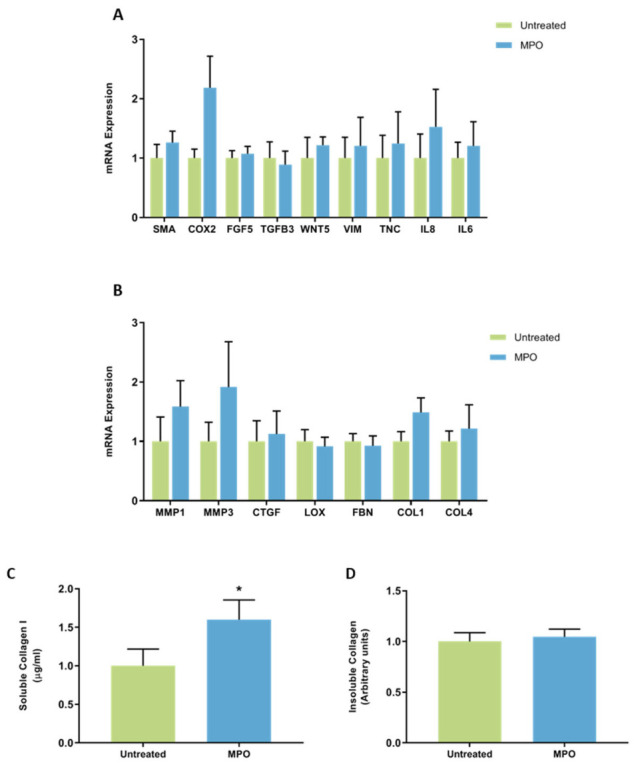
The effect of MPO on mRNA expression and collagen production in mammary fibroblasts. Messenger RNA expression of cancer-associated genes (**A**) and genes involved in extracellular matrix regulation and collagen production (**B**) measured by RT-PCR in human mammary fibroblasts following treatment with 5 μg/mL of MPO for 72 h. mRNA expression normalised to HPRT1 expression and presented as relative expression where the average for the untreated control is 1. Collagen production was measured using collagen 1 ELISA (**C**) and insoluble collagen measured by sirius red staining (**D**). Data presented as mean + SEM with statistical analysis performed using a linear mixed-effects model Tukey’s post hoc comparison. Statistical significance indicated by * when *p* < 0.05.

No significant differences in mRNA expression of CAF-related genes were observed in mammary fibroblasts treated with MPO (Figure 4A). No significant differences in mRNA expression of genes encoding genes involved in ECM regulation were observed when cells were treated with MPO (Figure 4B).

Collagen production was measured by ELISA to detect the production of soluble collagen I, and Sirius red dye to measure insoluble collagen fibres. Results were normalised so that collagen production for untreated controls was 1. Collagen I production was significantly increased in fibroblasts treated with MPO (1.61 ± 0.26 µg/mL) (Figure 4C) compared to controls (1 ± 0.22 µg/mL), and this increase was not dependant on the fibroblasts being from a woman with high or low MD (Figure 3C). No significant differences were observed in the production of collagen fibres in fibroblasts treated with MPO (Figure 4D).

#### The Effect of MPO on Gene Expression and Collagen Production by Mammary Fibroblasts from High and Low MD Breast Tissue

**Figure 5:**
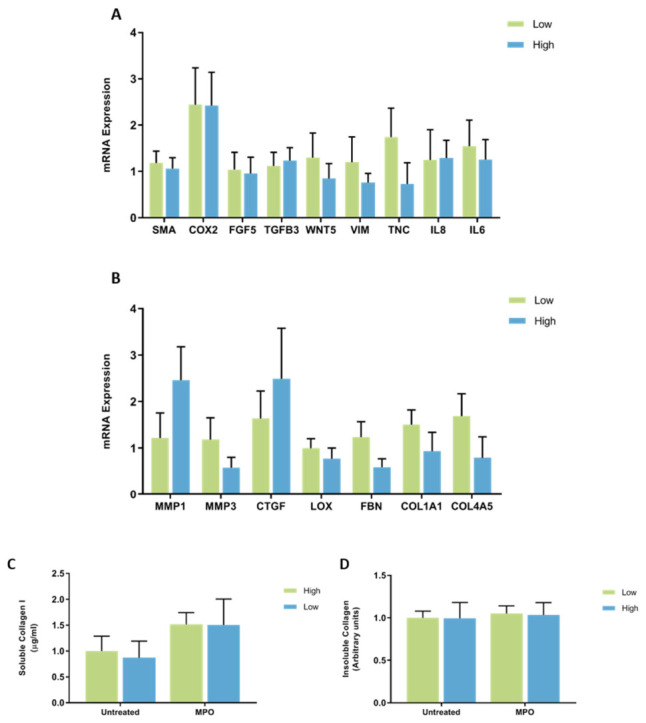
The effect of MPO on mRNA expression and collagen production in mammary fibroblasts isolated from women with high and low mammographic density. Messenger RNA expression of cancer-associated genes (**A**) and genes involved in extracellular matrix regulation and collagen production (**B**) measured by RT-PCR in human mammary fibroblasts from women with high (*n* = 7) and low (*n* = 9) mammographic density following treatment with 5 μg/mL of MPO for 72 h. mRNA expression normalised to HPRT1 expression and presented as relative expression where the average for untreated control is 1. Collagen production was measured using collagen 1 ELISA (**C**) and insoluble collagen measured by sirius red staining (**D**). Data presented as mean + SEM with statistical analysis performed using a linear mixed-effects model Tukey’s post hoc comparison.

When fibroblast samples were separated according to whether they came from a patient with high (*n* = 7) or low (*n* = 9) MD no differences in mRNA expression of CAF-related genes were observed between fibroblasts from high and low MD tissues when treated with MPO (Figure 5A). MPO did not have differential effects on mRNA expression of ECM regulatory genes from high MD fibroblasts compared to low MD fibroblasts (Figure 5B). No differences in soluble collagen I or insoluble collagen production were observed between fibroblasts from high and low MD tissues when treated with MPO (Figure 5C,D).

### 3.3. The Effect of EPO on ECM and CAF mRNA Expression, and Collagen Production by Mammary Fibroblasts

To investigate the effects on EPO and mammographic density on mammary fibroblasts, primary mammary fibroblasts were cultured for 72 h with 2.5 µg/mL of EPO in serum-free media (*n* = 16). mRNA expression of CAF genes and genes involved in ECM regulation were measured using quantitative real time PCR relative to the expression of the housekeeping gene *HPRT1* from the same patient. The results are expressed in arbitrary units as the data were normalised so that the average expression of each gene in untreated controls was equal to 1.

**Figure 6:**
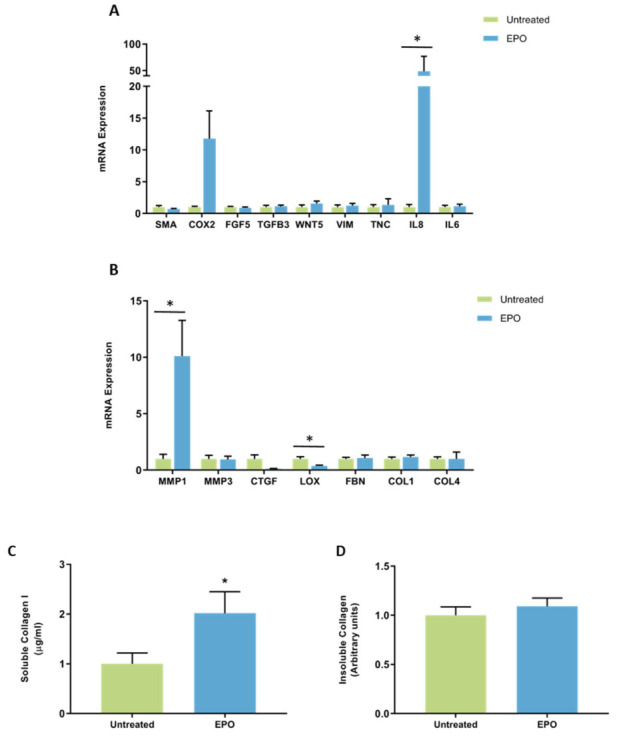
The effect of EPO on mRNA expression and collagen production in mammary fibroblasts. Messenger RNA expression of cancer-associated genes (**A**) and genes involved in extracellular matrix regulation and collagen production (**B**) measured by RT-PCR in human mammary fibroblasts following treatment with 2.5 μg/mL of EPO for 72 h. mRNA expression normalised to HPRT1 expression and presented as relative expression where the average for untreated controls is 1. Collagen production was measured using collagen 1 ELISA (**C**) and insoluble collagen measured by sirius red staining (**D**). Data presented as mean + SEM with statistical analysis performed using a linear mixed-effects model Tukey’s post hoc comparison. Statistical significance indicated by * when *p* < 0.05.

The expression of mRNA encoding *COX2* was significantly attenuated by treatment with EPO (11.76 ± 4.37 arbitrary units), with expression increased by over 10-fold compared to untreated controls (1 ± 0.15) (*p* < 0.05). Expression of *IL8* was also significantly increased by nearly 50-fold in fibroblasts treated with EPO (48.55 ± 28.50 arbitrary units), compared to untreated controls (1 ± 0.41 arbitrary units) (*p* < 0.05). No other changes in the expression of cancer-associated genes were observed in fibroblasts treated with EPO (Figure 6A).

Expression of mRNA encoding *MMP1* was significantly increased in fibroblasts treated with EPO (10.10 ± 3.17 arbitrary units) compared to untreated controls (1 ± 0.41 arbitrary units) (*p* < 0.01). There was a significant decrease in the expression of *LOX* in EPO-treated fibroblasts (0.39 ± 0.06 arbitrary units) compared to untreated controls (1 ± 0.19 arbitrary units) (*p* < 0.01) (Figure 6B). No other changes in the expression of genes involved in ECM regulation were observed in fibroblasts treated with EPO (Figure 3B).

Collagen production was measured by ELISA to detect the production of soluble collagen I and sirius red dye to measure insoluble collagen fibres. Results were normalised so that collagen production in untreated controls was equal to 1. Collagen I production was significantly increased in fibroblasts treated with EPO (2.04 ± 0.43 µg/mL) compared to untreated controls (1 ± 0.22 µg/mL) (*p* < 0.01) (Figure 6C). No significant differences were observed in the production of collagen fibres in fibroblasts treated with EPO (Figure 6D).

#### The Effect of EPO on Gene Expression and Collagen Production by Mammary Fibroblasts from High and Low MD Breast Tissue

**Figure 7:**
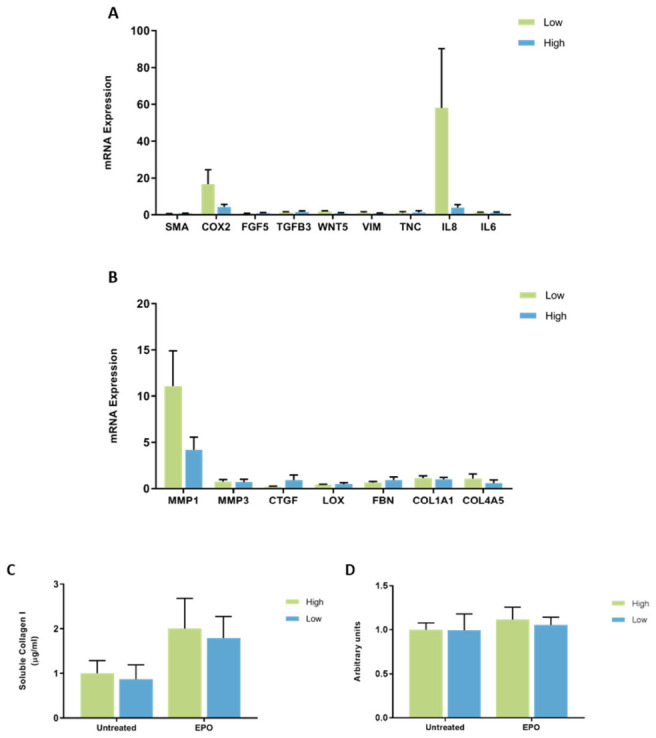
The effect of EPO on mRNA expression and collagen production in mammary fibroblasts isolated from women with high and low mammographic density. Messenger RNA expression of cancer-associated genes (**A**) and genes involved in extracellular matrix regulation and collagen production (**B**) measured by RT-PCR in human mammary fibroblasts from women with high (*n* = 7) and low (*n* = 9) mammographic density following treatment with 2.5 μg/mL of EPO for 72 h. mRNA expression normalised to HPRT1 expression and presented as relative expression where the average for untreated control is 1. Collagen production was measured using collagen 1 ELISA (**C**) and insoluble collagen measured by sirius red staining (**D**). Data presented as mean + SEM with statistical analysis performed using a linear mixed-effects model Tukey’s post hoc comparison.

When the results were separated according to the MD score of each patient sample, it was observed that there were no differences in the expression of cancer-associated genes between fibroblasts from high and low MD patient samples when treated with EPO (Figure 7A). No differences in the mRNA expression of ECM regulatory genes were observed between fibroblasts from high and low MD patient samples when treated with EPO (Figure 7B). No differences in soluble collagen I or insoluble collagen production were observed between fibroblasts from high and low MD tissues when treated with EPO (Figure 7C,D).

### 3.4. The Effect of TGFB on CAF and ECM mRNA Expression and Collagen Production by Mammary Fibroblasts

To investigate the effects on TGFB and mammographic density on mammary fibroblasts, primary mammary fibroblasts were isolated from human breast tissue and cultured for 72 h with 10 ng/mL of TGFB in serum-free media (*n* = 16). mRNA expression of cancer-associated genes and genes involved in extracellular matrix regulation were measured using quantitative real time PCR relative to the expression of the housekeeping gene *HPRT1* from the same patient. Results are expressed in arbitrary units as the data were normalised so that the average expression of each gene in untreated controls was equal to 1.

**Figure 8:**
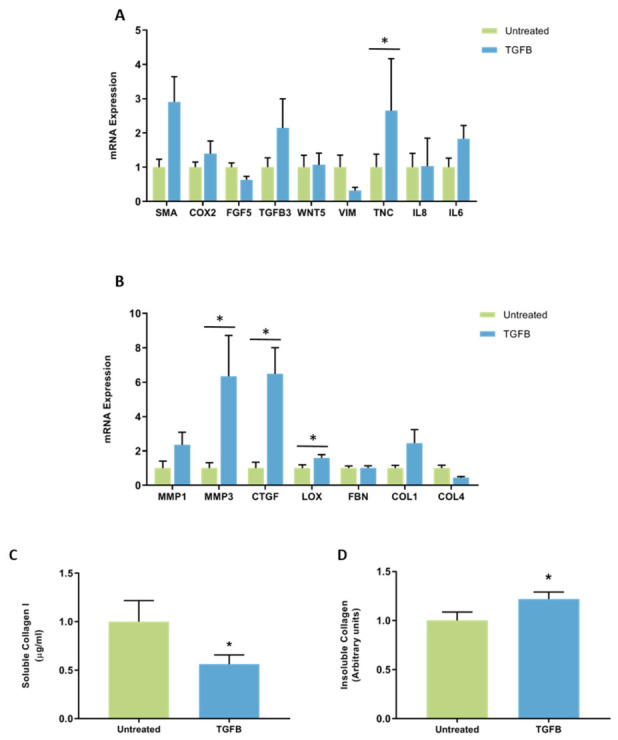
The effect of TGFB on mRNA expression and collagen production in mammary fibroblasts. Messenger RNA expression of cancer-associated genes (**A**) and genes involved in extracellular matrix regulation and collagen production (**B**) measured by RT-PCR in human mammary fibroblasts following treatment with 10 ng/mL of TGFB for 72 h. mRNA expression normalised to HPRT1 expression and presented as relative expression where the average for untreated control is 1. Collagen production was measured using collagen 1 ELISA (**C**) and insoluble collagen measured by sirius red staining (**D**). Data presented as mean + SEM with statistical analysis performed using a linear mixed-effects model Tukey’s post hoc comparison. Statistical significance indicated by * when *p* < 0.05.

Expression of mRNA encoding *TNC* was significantly increased in mammary fibroblasts treated with TGFB (2.64 ± 1.51 arbitrary units) compared to untreated controls (1 ± 0.38 arbitrary units). No other differences were observed in the expression of CAF genes in fibroblasts treated with TGFB (Figure 8A).

Expression of *MMP3* was increased by six-fold in fibroblasts treated with TGFB (6.34 ± 0.73 arbitrary units) compared to untreated controls (1 ± 0.32 arbitrary units) (*p* < 0.01). Expression of mRNA encoding *CTGF* was significantly increased in TGFB treated fibroblasts (6.48 ± 1.52 arbitrary units) compared to untreated controls (1 ± 0.34 arbitrary units) (*p* < 0.05). A small but significant increase in mRNA expression of *LOX* and in TGFB treated fibroblasts (1.58 ± 0.21 arbitrary units) was observed compared to untreated controls (1 ± 0.19 arbitrary units) (*p* < 0.01). A trending reduction in the expression of *COL4A5*, and a trending two-fold increase in *MMP1* expression was observed in fibroblasts treated with TGFB compared to untreated controls (*p* = 0.059, *p* = 0.074, respectively). No other changes in the expression of genes involved in extracellular matrix regulation were observed (Figure 8B).

Collagen production was measured by ELISA to detect the production of soluble collagen I and sirius red dye to measure insoluble collagen fibres. The results were normalised so that average collagen production in untreated controls was equal to 1. Collagen I production was significantly decreased in fibroblasts treated with TGFB (0.57 ± 0.1 arbitrary units) compared to untreated controls (1 ± 0.22 arbitrary units) (*p* < 0.01) (Figure 8C). There was a small but significant increase in insoluble collagen production in fibroblasts treated with TGFB (1.22 ± 0.07 arbitrary units) compared to untreated controls (1 ± 0.09 arbitrary units) (*p* < 0.01) (Figure 8D).

#### The Effect of TGFB on Gene Expression and Collagen Production by Mammary Fibroblasts from High and Low MD Breast Tissue

**Figure 9:**
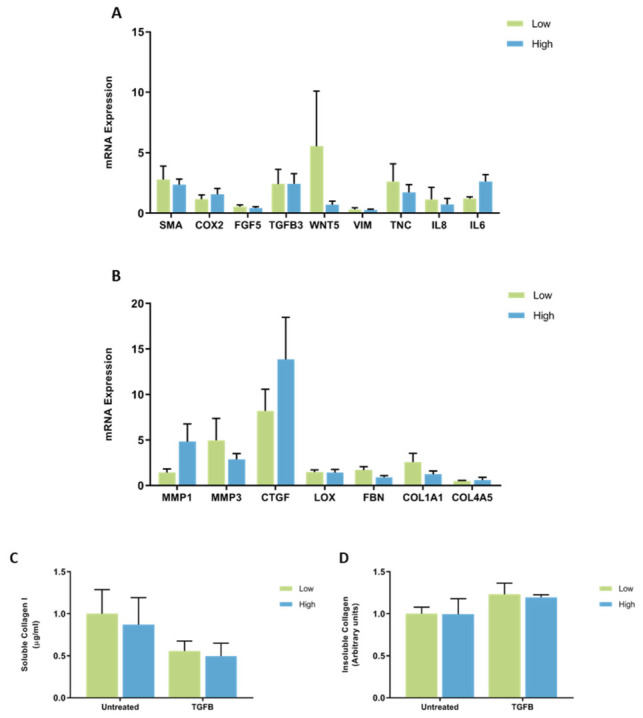
The effect of TGFB on mRNA expression and collagen production in mammary fibroblasts isolated from women with high and low mammographic density. Messenger RNA expression of cancer-associated genes (**A**) and genes involved in extracellular matrix regulation and collagen production (**B**) measured by RT-PCR in human mammary fibroblasts from women with high (*n* = 7) and low (*n* = 9) mammographic density following treatment with 10 ng/mL of TGFB for 72 h. mRNA expression normalised to HPRT1 expression and presented as relative expression where the average for untreated control is 1. Collagen production was measured using collagen 1 ELISA (**C**) and insoluble collagen measured by sirius red staining (**D**). Data presented as mean + SEM with statistical analysis performed using a linear mixed-effects model Tukey’s post hoc comparison.

When accounting for the patient’s MD score, there were no significant differences in the expression of CAF genes in fibroblasts from patients with high and low MD when treated with TGFB (Figure 9A). No significant differences were observed in the mRNA expression of ECM regulatory genes by fibroblasts from high and low MD patient samples when treated with TGFB (Figure 9B). No differences in soluble collagen I or insoluble collagen production were observed between fibroblasts from high and low MD tissues when treated with TGFB (Figure 9C,D).

### 3.5. The Effect of TNFA on CAF and ECM mRNA Expression and Collagen Production by Mammary Fibroblasts

To investigate the effects on tumour necrosis factor alpha (TNFA) and mammographic density on mammary fibroblasts, primary mammary fibroblasts were cultured for 72 h with 10 ng/mL of TNFA in serum-free media (*n* = 16). mRNA expression of CAF genes and genes involved in ECM regulation were measured using quantitative real time PCR relative to expression of housekeeping gene *HPRT1* from the same patient. Results are expressed in arbitrary units as the data were normalised so that the average expression of each gene in untreated controls was equal to 1.

**Figure 10:**
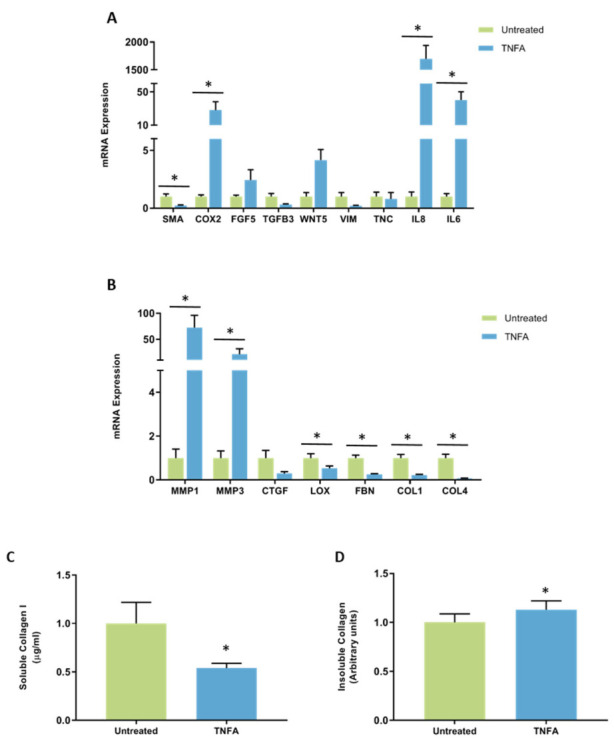
The effect of TNFA on mRNA expression and collagen production in mammary fibroblasts. Messenger RNA expression of cancer-associated genes (**A**) and genes involved in extracellular matrix regulation and collagen production (**B**) measured by RT-PCR in human mammary fibroblasts following treatment with 10 ng/mL of TNFA for 72 h. mRNA expression normalised to HPRT1 expression and presented as relative expression where the average for untreated control is 1. Collagen production was measured using collagen 1 ELISA (**C**) and insoluble collagen measured by sirius red staining (**D**). Data presented as mean + SEM with statistical analysis performed using a linear mixed-effects model Tukey’s post hoc comparison. Statistical significance indicated by * when *p* < 0.05.

A significant decrease in the mRNA expression of *SMA* was observed in TNFA treated fibroblasts (0.22 ± 0.05 arbitrary units) compared to untreated controls (1 ± 0.23 arbitrary units) (*p* < 0.05). The expression of mRNA encoding *COX2* was significantly increased in TNFA-treated fibroblasts (27.98 ± 10.15 arbitrary units) compared to untreated controls (1 ± 0.15 arbitrary units) (*p* < 0.01). The expression of mRNA encoding *IL8* and *IL6* were highly increased in TNFA-treated fibroblasts (1701.3 ± 237.8 and 40.29 ± 9.83 arbitrary units, respectively) compared to untreated controls (1 ± 0.40 and 1 ± 0.27 arbitrary units, respectively) (*p* < 0.001 for both genes). A four-fold trending increase in expression of *WNT5* was observed in TNFA-treated fibroblasts, though this was not significant (*p* = 0.051). No other differences in CAF gene expression were observed (Figure 10A).

There were also strong effects of TNFA on expression of genes involved in regulation of the extracellular matrix. The most potent effects were on the expression of *MMP1* and *MMP3*, which were significantly increased in TNFA-treated fibroblasts (72.43 ± 23.72 and 21.18 ± 10.57 arbitrary units, respectively) compared to untreated controls (1 ± 0.41 and 1 ± 0.32 arbitrary units, respectively) (*p* < 0.001 for both genes). A significant decrease in the expression of *LOX* and *FBN* was observed in TNFA-treated fibroblasts (0.53 ± 0.11 and 0.25 ± 0.04 arbitrary units, respectively) compared to untreated controls (1 ± 0.19 and 1 ± 0.13, respectively) (*p* < 0.01). Both collagen genes *COL1A1* and *COL4A5* were decreased in TNFA-treated fibroblasts (0.22 ± 0.03 and 0.07 ± 0.01 arbitrary units, respectively) compared to untreated controls (1 ± 0.16 and 1 ± 0.17 arbitrary units, respectively) (*p* < 0.01) (Figure 10B).

Collagen production was measured by ELISA to detect the production of soluble collagen I and sirius red dye to measure insoluble collagen fibres. Results were normalised so that average collagen production in untreated controls was equal to 1. Collagen I production was significantly decreased in fibroblasts treated with TNFA (0.54 ± 0.06 arbitrary units) compared to untreated controls (1 ± 0.22 arbitrary units) (Figure 10C). A small but significant increase in Sirius red staining was observed in TNFA-treated fibroblasts (1.13 ± 0.09 arbitrary units) compared to untreated controls (1 ± 0.09 arbitrary units) (Figure 10D).

#### The Effect of TNFA on Gene Expression and Collagen Production by Mammary Fibroblasts from High and Low MD Breast Tissue

**Figure 11:**
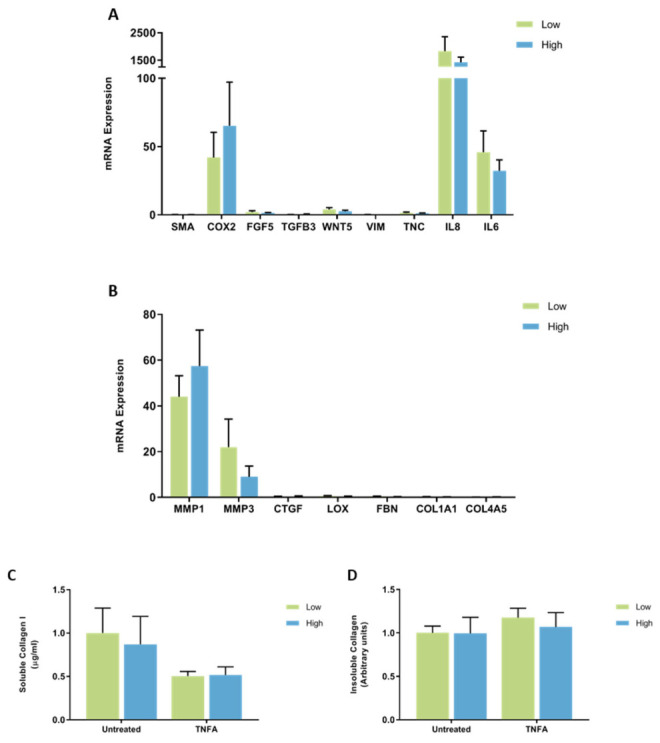
The effect of TNFA on mRNA expression and collagen production in mammary fibroblasts isolated from women with high and low mammographic density. Messenger RNA expression of cancer-associated genes (**A**) and genes involved in extracellular matrix regulation and collagen production (**B**) measured by RT-PCR in human mammary fibroblasts from women with high (*n* = 7) and low (*n* = 9) mammographic density following treatment with 10 ng/mL of TNFA for 72 h. mRNA expression normalised to HPRT1 expression and presented as relative expression where the average for untreated control is 1. Collagen production was measured using collagen 1 ELISA (**C**) and insoluble collagen measured by sirius red staining (**D**). Data presented as mean + SEM with statistical analysis performed using a linear mixed-effects model Tukey’s post hoc comparison.

When the results were separated according to the MD score of each patient sample, no differences in the expression of CAF-related genes were observed between fibroblasts from breast tissue with high or low MD when treated with TNFA (Figure 11A). There were no differences in the expression of genes involved in ECM regulation between fibroblasts from high or low MD patient samples when treated with TNFA (Figure 11B). There was no difference in collagen I production or insoluble collagen production between fibroblasts from breast tissue with high or low MD when treated with TNFA (Figure 11C,D).

## 4. Discussion

The underlying biological mechanisms that contribute to mammographic density and the associated breast cancer risk are largely unknown. As fibroblasts are a prominent cellular component of the mammary stroma, which is highly abundant in tissue with high mammographic density, it was hypothesised that they play a role in the development of mammographic density and breast cancer risk. This study sought to explore whether fibroblasts from the breast tissue of high MD exhibited altered responses to immune stimuli in terms of gene expression and extracellular matrix production, compared to fibroblasts from breast tissue with low MD. The results of this study suggest that while fibroblasts do respond to immune stimuli and are likely to be a critical cell type within the high MD microenvironment, fibroblasts isolated from high MD tissue are not inherently different in their behaviour in vitro compared to fibroblasts isolated from low MD tissue.

### 4.1. Mammary Fibroblasts Isolated from High MD Tissue Are Not Inherently Different from Those Isolated from Low MD Tissue

High MD breasts exhibit high abundance of stromal fibroblasts; however, previous studies have not investigated the potential of fibroblasts as drivers of MD. It was hypothesised that fibroblasts from high MD breast tissue may exhibit altered gene expression like that of CAFs that promote a more favourable environment for tumour development or have greater expression of genes involved in the production of the ECM and in this way act as drivers of mammographic density. However, the results here suggest that fibroblasts from high MD breasts are not inherently different from those from low MD breasts as there were no differences in ECM or CAF gene expression or collagen production between fibroblasts from breast tissue with high or low MD. Therefore, fibroblasts alone are unlikely to be driving mammographic density through their inherent fibrotic activities or contributing to breast cancer risk through greater expression of CAF genes. In addition, when these fibroblasts were stimulated with immune proteins and cytokines, there were still no differences in gene expression or collagen production between fibroblasts from high or low density breast tissue. This suggests that fibroblasts isolated from high density breast tissue do not have different responsiveness to immune stimulus compared to fibroblasts from low density breast tissue. However, these studies are subject to limitations and only measured a select subset of gene and protein expressions. Measures of other genes and proteins related to fibroblast functions could reveal differences between high and low MD fibroblasts.

### 4.2. Modulation of Extracellular Matrix Regulation and Collagen Production in Mammary Fibroblasts by Peroxidase Enzymes

Production of soluble collagen I was significantly increased in primary mammary fibroblasts when treated with myeloperoxidase or eosinophil peroxidase. However, there was no increase in mRNA expression of *COL1A1* in response to myeloperoxidase or eosinophil peroxidase. This is consistent with previous studies that reported that peroxidase enzymes stimulate the production of collagen I in fibroblasts derived from various tissue sites, as well as the production of collagen IV in vitro. This suggests that peroxidase enzymes have important roles in regulating the extracellular matrix at the post-translational level. However, our study found no change in collagen fibre staining in response to peroxidase enzymes. Therefore, peroxidase enzymes may be involved in intracellular post-translational modifications prior to extracellular collagen fibril assembly.

Interestingly, eosinophil peroxidase significantly increased the mRNA expression of *COX2*. *COX2* is the gene encoding cyclooxygenase, an enzyme that catalyses the production of prostaglandins, and is expressed during inflammation [23]. COX2 has been implicated in mammographic density and breast cancer risk in previous studies. In a study by Yang et al. in 2009, ChIP-seq analysis of normal breast tissue demonstrated that expression of COX2 was increased in tissues from women with high mammographic density, and this was further confirmed by immunohistochemistry [24]. In women, expression of *COX2* has been found to be upregulated in 56% of mammary tumours, and COX2 inhibitors have been trialled as chemoprevention therapies [25,26]. Therefore, peroxidase enzymes may play a role in mammographic density and the associated breast cancer risk through promoting the production of collagen 1 by mammary fibroblasts and also in the induction of COX2 expression.

### 4.3. TGFB1 Signalling Modulates Extracellular Matrix Regulation and Collagen Production in Mammary Fibroblasts

Our studies found that mammary fibroblasts treated with TGFB1 displayed increased mRNA expression of genes involved in ECM regulation such as *MMP3*, *CTGF* and *LOX*, as well as alterations in collagen production. TGFB is a key regulator of fibrosis and is upregulated in most fibrotic diseases through excessive production of ECM components, including collagen [27].

Connective tissue growth factor (*CTGF*) was highly expressed in fibroblasts treated with TGFB. CTGF is expressed by fibroblasts following activation by TGFB [28], however it has also been shown that CTGF alone can induce production of collagen by fibroblasts [29,30]. Studies have suggested that CTGF, through TGFB signalling, is a key regulator of fibrosis [31]. In mice, CTGF can drive fibrosis in the lungs and the inhibition of CTGF signalling can prevent liver fibrosis in rats [32,33]. Overexpression of CTGF has been linked to increased tumour size in breast cancer and together with MMPs can contribute to the tissue remodelling required for metastasis and angiogenesis [34,35].

While the production of soluble collagen I was decreased in TGFB-treated fibroblasts, an increase in sirius red staining for insoluble collagen fibres was observed in these cells. This may be the result of an increase in the expression of *LOX,* which encodes the enzyme lysyl oxidase required to maintain the structural integrity of collagen fibres by facilitating the covalent cross-linking of collagen and elastin into insoluble fibres, and protecting collagen fibres from being degraded [17,36]. The expression of LOX is upregulated in several fibrotic diseases associated with an accumulation of collagen and ECM components, such as pulmonary fibrosis, skin tissues, hepatic fibrosis and cardiac fibrosis [37,38,39,40,41]. These fibrotic effects of LOX are mediated through interactions with TGFB [39,40]. LOX has also been implicated in breast cancer risk. In a study by Levental and colleagues, overexpression of LOX in the mammary glands of MMTV-Neu mice lead to increased tumour growth with more invasive properties. Similarly, inhibition of LOX expression resulted in reduced ECM stiffness in the mammary gland, preventing fibrosis and reduced tumour susceptibility [42].

Treatment with TGFB induced an increase in the expression of *MMP3*, a matrix metalloproteinase that is commonly thought to degrade components of the extracellular matrix [43]. However, studies have reported that MMP3 can promote the transformation of latent TGFB to its active form and can therefore promote fibrotic processes [44]. These functions of MMP3 are poorly understood, and further investigation is required to understand the role of MMP3 in fibrotic processes and TGFB signalling.

Our study demonstrates that TGFB can induce the expression of ECM genes with pro-fibrotic activities and increase the production of insoluble collagen fibres. This suggests that TGFB could contribute to the increased stroma and collagen deposition seen in high density breast tissue [1,45]. However, previous studies in women with high mammographic density have found a decrease in TGFB signalling in breast tissue samples using ChIP-seq [24]. Therefore, further studies are required to understand the role of TGFB in mammographic density.

### 4.4. TNFA Signalling and Fibroblast Function

Our studies investigating the effects of TNFA on mammary fibroblast function demonstrated that TNFA has profound effects on mammary fibroblast gene expression. TNFA significantly decreased the mRNA expression of the genes involved in ECM deposition, including *LOX*, *FBN*, *COL1A1* and *COL4A5*, as well as decreased production of soluble collagen I. This suggests that TNFA is imposing anti-fibrotic effects on mammary fibroblasts. Further, TNFA increased the expression of *MMP1* and *MMP3*, which encode matrix metalloproteinases that have the primary function of degrading collagen and other ECM components [43]. These MMPs have been identified for use as potential therapeutics for fibrotic diseases due to their capacity for breaking down the excessive deposits of ECM components [44]. These results are consistent with studies that investigated the role of TNFA in the skin, where TNFA induced expression of both *MMP1* and *MMP3* in skin tissue explants and accelerated the degradation of collagen 1 [46]. Other studies have also reported that TNFA inhibits the production of collagen 1 and promotes the expression of collagenases such as MMPs [47,48]. This suggests that inflammation driven by TNFA may not promote the increased collagen and pro-fibrotic environment seen in high MD breast tissue. However, an increase in insoluble collagen deposition was observed in TNFA-treated fibroblasts and there are some studies that suggest that TNFA can promote collagen deposition [18]. Therefore, the effects of TNFA on collagen production in the breast require further investigation to be understood.

TNFA induced high expression of inflammatory genes *IL6*, *IL8* and *COX2* in mammary fibroblasts. In the breast, inflammation mediated by TNFA, IL6 and IL8 can favour a pro-tumorigenic environment and promote tumour initiation, growth, and progression [49]. As previously discussed, COX2 expression is increased in the breast tissue of women with high MD [24,50]. In mice with high collagen content in the mammary gland, pharmacological COX2 inhibition is associated with a decrease in tumour development and metastasis [51].

Although TNFA promoted the expression of inflammatory genes that could promote a pro-tumour environment, it did not induce a CAF-like phenotype, as one of the standard markers of CAFs, *SMA*, was decreased in TNFA treated fibroblasts [13]. Therefore, TNFA may contribute to the inflammation associated with MD and breast cancer risk, but more studies are required.

### 4.5. Limitations

The experiments performed in this study have several limitations that affect the results and the subsequent conclusions drawn from them. Specifically, low sample sizes, inter-patient variability and the specific culture methods used could have affected the results obtained.

These studies were limited by the availability of patient tissues of high and low mammographic density. Most women have breasts in the mid-range of density, either scattered density or heterogeneously dense, so there were a limited number of samples obtained from the extreme ends of the spectrum of mammographic density. A great sample size could allow for the confounding of factors that can influence mammographic density such as age or BMI and potentially reduce the variability in the results. Consideration of age in patient data is important for mammographic density research, as density is known to decrease with age [52]. However, age-related alterations in the mammary stroma and immune microenvironment may foster more inflammatory and pro-tumorigenic signalling [53].

The treatment conditions in this experiment were limited and the 72 h time point was investigated, which may not accurately represent how mammary fibroblasts respond to these immune factors. These experiments are more reflective of an acute response to stimulation, and the environment that contributes to mammographic density may be a more chronic response whereby the fibroblasts are more affected by longer exposure to stimuli such as immune signalling proteins. Analysing the mRNA expression and collagen production from mammary fibroblasts in response to immune proteins would be more meaningful if RNA had been collected at multiple time points. The experiments performed in this chapter were in two-dimensional culture and may not accurately represent how fibroblasts act within human tissues. Future studies may consider three-dimensional culture models or tissue explants to further analyse cellular functions in MD. The concentration of immune proteins used in these experiments were the lowest concentration that yielded the highest affect as determined by previous studies. While this may yield the greatest effect of the treatment, it may not be the physiologically relevant concentration found in human tissues [16,54,55]. Therefore, analysis of fibroblasts’ responses to immune proteins at various concentrations would be meaningful. While studies of gene expression and collagen production are valuable, there are many other features of mammary fibroblasts that could be measured to characterise their behaviour in high and low density breast tissues such as genome-wide studies, protein expression, migration and interactions with other cells.

The experiments described in this paper are subject to inter-patient variability in gene expression analyses from primary mammary fibroblasts. There was a very large degree of variability in the expression of some genes that suggest other unknown factors apart from MD have a great influence on gene expression in mammary fibroblasts. While these studies examine very specific subsets of genes in isolated cells, it is important to note that the cells were derived from tissues within a full body system that have been exposed to environmental, lifestyle and epigenetic factors that may influence the baseline functions of the cells within each patient.

## 5. Conclusions

By utilising primary mammary fibroblasts from patients with high and low mammographic density, we have shown that fibroblasts isolated from high density breast tissue are not inherently different from those isolated from low density breast tissue regarding their gene expression and collagen production in vitro. However, the sample size was small and there was a large amount of variability between patients. We have also demonstrated that peroxidase enzymes and other immune regulatory proteins such as TGFB and TNFA can have profound effects on the behaviour of mammary fibroblasts and can induce pro-fibrotic gene expression and collagen production and promote an inflammatory microenvironment that might be more favourable for tumour development. The roles of peroxidases and immune proteins in mammographic density are complex, and further studies are required to elucidate the cellular and molecular interactions that drive breast cancer risk.

## Figures and Tables

**Table 1:** Patient characteristics.

	Low MD (*n* = 9)	High MD (*n* = 7)
Age (median; range)	48 years (40–73)	32 years (20–52)
Body mass index (median; range)	31.2 kg/m^2^ (21.8–42.5)	23.6 kg/m^2^ (18.5–28.4)
Family history (*n*; %)	2 (22)	2 (29)
Breast cancer (*n*; %)	6 (67)	1 (14)
Parous (*n*; %)	8 (89)	6 (86)

## Data Availability

Data available upon request.
